# Low-Power Branch CNN Hardware Accelerator with Early Exit for UAV Disaster Detection Using 16 nm CMOS Technology

**DOI:** 10.3390/s25154867

**Published:** 2025-08-07

**Authors:** Yu-Pei Liang, Wen-Chin Chao, Ching-Che Chung

**Affiliations:** Department of Computer Science and Information Engineering, Advanced Institute of Manufacturing with High-Tech Innovations, National Chung Cheng University, Chia-Yi 621301, Taiwan; ypliang@cs.ccu.edu.tw (Y.-P.L.); chao860204@gmail.com (W.-C.C.)

**Keywords:** unmanned aerial vehicles (UAVs), disaster detection, neural networks, quantization, fixed-point arithmetic, real-time systems, early-exit mechanism, digital circuits

## Abstract

This paper presents a disaster detection framework based on aerial imagery, utilizing a Branch Convolutional Neural Network (B-CNN) to enhance feature learning efficiency. The B-CNN architecture incorporates branch training, enabling effective training and inference with reduced model parameters. To further optimize resource usage, the framework integrates DoReFa-Net for weight quantization and fixed-point parameter representation. An early exit mechanism is introduced to support low-latency, energy-efficient predictions. The proposed B-CNN hardware accelerator is implemented using TSMC 16 nm CMOS technology, incorporating power gating techniques to manage memory power consumption. Post-layout simulations demonstrate that the proposed hardware accelerator operates at 500 MHz with a power consumption of 37.56 mW. The system achieves a disaster prediction accuracy of 88.18%, highlighting its effectiveness and suitability for low-power, real-time applications in aerial disaster monitoring.

## 1. Introduction

In recent years, unmanned aerial vehicles (UAVs) have attracted considerable attention as a remote sensing platform across various application domains, including agricultural monitoring [1], search and rescue operations [2], and traffic management [3]. Traditional cloud computing architectures require data transmission between terminal devices and remote servers, which introduces challenges such as data security vulnerabilities, privacy concerns, and network latency. UAVs address these issues by autonomously executing computational and processing tasks, thereby enabling more efficient and reliable detection and recognition.

Moreover, UAVs are widely utilized in disaster detection. They offer efficient monitoring capabilities, enabling rapid coverage of large areas and facilitating timely acquisition of disaster overviews. This capability supports swift situational awareness and the implementation of appropriate response measures. In addition, UAVs are adept at navigating complex geographical and topographical conditions, including mountainous regions, bodies of water, and urban environments, thus demonstrating significant potential in disaster monitoring applications. Notably, UAVs are equipped with high-resolution cameras that allow for the acquisition of detailed imagery, thereby enhancing the accuracy of disaster assessment. For instance, UAV-mounted cameras can distinctly capture submerged structures during floods [4], the formation of landslides after slope collapses [5], and burning areas in wildfires [6]. Finally, UAVs can promptly gather and transmit disaster-related information to command centers, contributing to the mitigation of disaster-induced losses.

The limited flight endurance of UAVs constrains their capacity for continuous monitoring, spatial coverage, and high-resolution observation, thereby posing significant challenges to effective disaster response and loss mitigation. For instance, a long-range DJI drone [7] equipped with a 4280 mAh battery typically achieves a flight duration of 28 min. When loaded with a 5 V, 5 W Jetson Nano module [8], its flight time decreases drastically to approximately 3.7 min, reducing by about 87%, and this does not even consider the impact of weight. In contrast, when carrying a 40 mW Application-Specific Integrated Circuit (ASIC), the drone can maintain a flight time of 21 min, representing only a 25% reduction. These comparisons underscore the critical need for developing low-power chips to extend UAV flight time, which is essential for effectively executing disaster detection missions.

Additionally, the aforementioned applications are limited to the detection of individual events, resulting in UAVs being unable to effectively adapt to different environments. This limitation hinders the development of UAVs from disaster prevention and management. Hence, the creation of AIDER [9] dataset entailed the manual collection of images for four disaster classes: Fire, Flood, Collapsed Building, Traffic Accidents, along with a category representing the Normal class.

Prior studies have explored architectural and training optimizations to enhance model performance. For example, in Ref. [10], the use of atrous convolutions enables multi-scale feature extraction by enlarging the receptive field without increasing parameter count. In Ref. [11], data augmentation techniques (e.g., rotation, flipping, intensity variation) improve model generalization, while different layer configurations are evaluated to balance accuracy and complexity. In Ref. [12], EfficientNet is adopted as a backbone model, employing width scaling and attention mechanisms to focus on salient features while maintaining a lightweight structure.

However, many existing approaches emphasize accuracy at the expense of computational complexity and energy efficiency, thereby limiting their feasibility for deployment on power-constrained UAV platforms. To address this limitation, an energy-efficient and lightweight disaster classification model is proposed, suitable for real-time deployment on UAVs while maintaining high accuracy and full functional capability.

In this paper, a lightweight convolutional neural network (CNN) model is proposed for disaster recognition. The model incorporates a hierarchical classification framework based on coarse and fine category levels to enhance classification accuracy. In addition, an early exit mechanism is employed to substantially reduce computational overhead. For hardware implementation using the TSMC 16 nm CMOS process, weight and activation quantization techniques are applied to reduce memory space demand. These strategies collectively improve the feasibility and efficiency of deploying neural networks on UAVs for disaster classification tasks.

The main contributions of this paper can be summarized as follows:

1. A hierarchical classification strategy is integrated to differentiate between coarse- and fine-grained disaster categories. This approach enhances the semantic richness and interpretability of the learned features, allowing the model to achieve comparable or superior accuracy with a reduced number of parameters.
2. A lightweight convolutional neural network incorporating an early-exit mechanism is designed to enable dynamic inference based on prediction confidence. This significantly reduces computational demands and energy consumption, facilitating low-latency and energy-efficient disaster recognition suitable for deployment on UAV platforms.
3. The proposed model is designed with resource efficiency in mind, minimizing memory usage and computational overhead to enable deployment on power-constrained UAV hardware. This ensures practical feasibility while maintaining reliable performance in real-world disaster monitoring scenarios.

The remainder of the paper is organized as follows: Section 2 presents an overview of the related work on model quantization. Section 3 shows the architecture of the proposed B-CNN. Section 4 describes the hardware implementation of the proposed B-CNN. Section 5 provides the experimental results, while the conclusion is presented in Section 6.

## 2. Related Work

Recent advances in model compression and efficient deep learning have promoted quantization as a key strategy for deploying neural networks on resource-constrained devices. In this section, existing methods are categorized into three groups: weight quantization techniques, activation quantization and function optimization, and architectural innovations that enhance inference efficiency.

### 2.1. Weight Quantization Techniques

Traditional deep neural networks rely on 32-bit floating-point precision, offering high accuracy at the cost of computational and memory demands. To alleviate this, many studies have explored reducing the bit-width of weights. For instance, Binary Neural Networks (BNNs) [13] restrict weights to +1 and −1, drastically lowering memory usage and computational cost. However, the representational capacity is significantly limited, often resulting in noticeable accuracy degradation. Ternary Weight Networks (TWNs) [14] allow weights of +1, 0, and −1, offering improved accuracy and a trade-off between model sparsity and performance. Nevertheless, both BNNs and TWNs face challenges in maintaining high accuracy, particularly for complex tasks such as disaster recognition.

### 2.2. Activation Quantization and Function Optimization

Activation functions such as ReLU are commonly used due to their simplicity, but their unbounded outputs can lead to quantization inefficiencies. To address this, Parameterized Clipping Activation (PACT) [15] introduces a learnable clipping parameter *α*, which adaptively bounds activations during training, effectively reducing quantization error. DoReFa-Net [16] further generalizes this idea by applying quantization to both weights and activations at arbitrary bit-widths. It introduces a general *k*-bit quantization function, as shown in Equation (1).

(1) $${quantized}_{k}\left(x\right)=\frac{1}{2^{k}-1}round\left(\left(2^{k}-1\right)x\right)$$

For weight quantization, weights are first constrained to the [−1, 1] range using the hyperbolic tangent function, then normalized and quantized as shown in Equation (2).

(2) $$f_{w}^{k}\left(r\right)=2{quantize}_{k}\left(\frac{\tanh\left(r\right)}{2max(|\tanh\left(r\right)|)}+\frac{1}{2}\right)-1$$

### 2.3. Architectural Innovations for Efficient Inference

Beyond quantization, architectural strategies have emerged to improve efficiency. Branch Convolutional Neural Networks (B-CNNs) [17] introduce hierarchical outputs aligned with semantic label structures, enabling both coarse- and fine-grained classification with intermediate supervision. This enhances interpretability while reducing computational overhead. BranchyNet [18] extends this concept with early-exit mechanisms that allow inference to terminate once confidence thresholds are met, reducing latency and energy consumption. However, these architectures require careful design to avoid performance bottlenecks and to ensure early branches still provide reliable predictions.

### 2.4. Summary

In summary, previous research has predominantly concentrated on software-level optimizations and neural network architecture design, while hardware solutions addressing these issues remain relatively scarce. As mentioned earlier, deploying existing embedded platforms (e.g., Jetson Nano) that support deep learning inference on UAVs significantly increases battery consumption, thus shortening flight duration. On the other hand, relying on cloud-based inference requires stable, high-bandwidth communication networks, which can often be unreliable or unavailable in disaster-affected areas. To address these challenges, this work proposes a customized ASIC solution specifically designed for UAV-based disaster recognition, aiming to enable real-time, energy-efficient inference under conditions of limited power consumption and constrained communication.

## 3. The Proposed B-CNN Architecture

### 3.1. B-CNN Architecture Overview

This paper presents a branch CNN-based approach, referred to as B-CNN, for low-power disaster detection, suitable for real-time classification. As previously noted, the AIDER dataset contains various disaster scenarios captured from aerial imagery and is thus adopted in this work as a case for method evaluation. Specifically, a total of 6433 aerial images were used. After random shuffling, 80% of the data (5147 images) were allocated for model training and the remaining 20% (1286 images) for testing. Notably, while the dataset provides diverse scenes and multiple disaster types, it primarily focuses on event classification and does not explicitly annotate environmental conditions such as fog, rain, or nighttime lighting. Detailed information about the training set and test set in each class is provided in Table 1.

**Figure 1:**
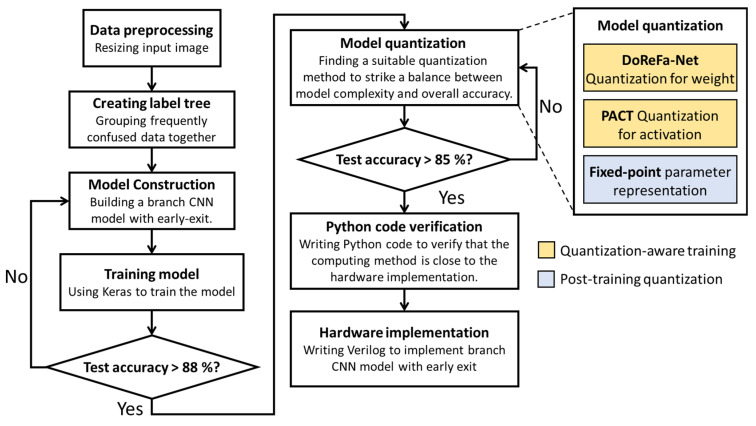
Flowchart for designing the software B-CNN model.

Figure 1 depicts the workflow for constructing the proposed branch CNN model with an early-exit mechanism. As illustrated, following image preprocessing, the first step involves constructing a label tree for the dataset. This tree clusters frequently confused classes together to facilitate hierarchical prediction. After completing data preprocessing and label tree construction, a suitable neural network architecture is selected through iterative experimentation. Given that images captured by drones are predominantly of the normal class, the early-exit mechanism can be effectively applied at the coarse prediction stage to conserve energy and reduce computational overhead.

Subsequently, the model is implemented and trained using Keras until a satisfactory test accuracy is achieved. Upon completion of model construction, a series of quantization techniques were implemented to enable deployment on edge devices. Given the limited computational and storage resources of edge hardware, reducing memory consumption and computational complexity is crucial. To address these constraints, DoReFa-Net was applied for weight quantization, while PACT was adopted for activation quantization. Both approaches are quantization-aware methods that integrate quantization into the training phase and retrain the model, thus minimizing accuracy degradation caused by quantization. Following quantization-aware training, all model parameters are converted into fixed-point representations, offering better hardware compatibility and significantly reducing implementation costs. Notably, this procedure can be considered as post-training quantization-aware processing. As illustrated in Figure 1, this strategy achieves an effective balance between model efficiency and accuracy, with only minimal accuracy degradation. Finally, Python 3.10.x code is employed to validate the consistency between software-based and hardware-based computations, marking the completion of the software implementation phase.

In selecting an appropriate image preprocessing strategy, it is essential to evaluate its impact on model accuracy and computational resource consumption. Since the images in the dataset vary in size, all inputs are initially resized to 128 × 128 pixels to balance model complexity and predictive performance. A preliminary five-layer CNN model is constructed to facilitate the evaluation of various preprocessing approaches. Subsequently, as shown in Table 2, experiments are conducted using different image resizing strategies to assess their effects on classification accuracy.

The results presented in Table 2 demonstrate the substantial impact of the nearest-neighbor interpolation method on classification accuracy, with a marked decline observed when images are scaled to 32 × 32 pixels. In contrast, the other three interpolation methods yield superior performance. Although area and bicubic interpolation methods achieve slightly higher accuracy than bilinear interpolation, they impose significantly greater computational complexity. Therefore, considering both computational efficiency and classification accuracy, the proposed architecture adopts bilinear interpolation and resizes input images to 64 × 64 pixels for subsequent model training and evaluation.

Prior to constructing the branch CNN model, it is necessary to establish a label tree for the dataset by grouping classes that are frequently misclassified. The dataset comprises five classes, with the Normal class isolated to enable its use within the early-exit mechanism. Initially, a compact five-layer neural network is developed and trained on the remaining classes intended for hierarchical classification.

As shown in Table 3, the class most frequently confused with Traffic Accidents is Collapsed Building, indicating a strong tendency for misclassification between these two categories. Consequently, they are grouped together to form a subclass named Accident. Similarly, a high degree of mutual confusion is observed between Fire and Flood, leading to their grouping into a subclass named Disaster.

**Figure 2:**
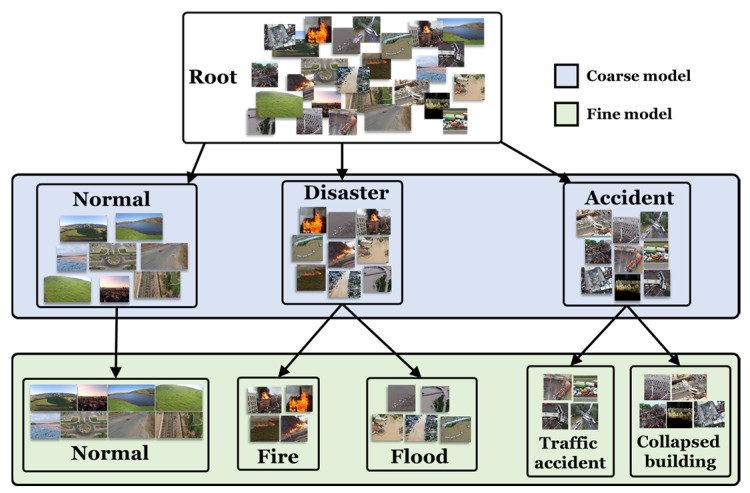
The label tree corresponding to the dataset (all the figures are from the AIDER dataset) [9].

To better understand the proposed model, the final label tree for the dataset is constructed, as illustrated in Figure 2. Based on this hierarchical structure, the branch CNN model produces both coarse- and fine-grained predictions for each input image. For example, when processing an image depicting a fire, the model first outputs a coarse prediction of Disaster, followed by a fine prediction of Fire. This hierarchical prediction strategy enhances the semantic richness and interpretability of the model’s outputs, facilitates more effective feature representation learning, and ultimately improves overall classification accuracy.

**Figure 3:**
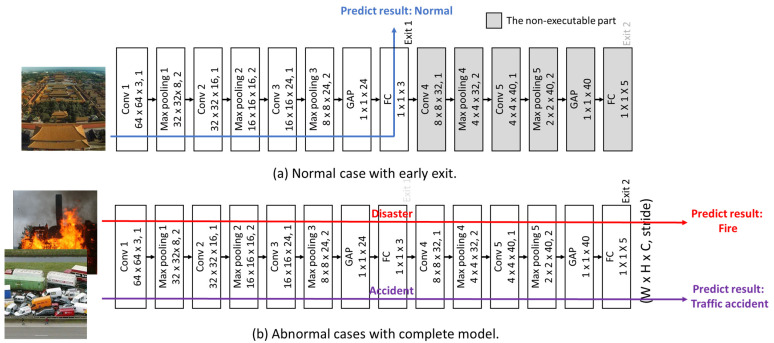
The proposed model with example input cases. The numbers inside each layer indicate the dimensions in the format (width × height × channels, stride).

The feature map sizes for each layer of the proposed branch CNN model are presented in Figure 3. To maintain the spatial dimensions of the feature maps after convolution, zero padding is employed. However, this may cause the receptive field center of the kernel to overlap with edge data points, potentially affecting boundary information. A stride of 1 is used in the convolution operations to enable the extraction of fine-grained features, albeit at the cost of increased computational complexity. Consequently, pooling is performed with a stride of 2 to progressively reduce the size of the subsequent feature maps, thereby decreasing memory usage and computational overhead. In addition, as shown in Figure 3, normal cases exit and produce prediction results after Convolution Layer 3, while abnormal cases are processed through the full model and exit after Convolution Layer 5.

Furthermore, Table 4 illustrates the accuracy differences between the branch CNN model with and without batch normalization. Although batch normalization introduces additional parameters and thus imposes greater hardware overhead, it offers notable benefits in terms of accelerating training and enhancing model stability. The inclusion of batch normalization results in an approximate 3% to 4% improvement in accuracy. Therefore, incorporating batch normalization into the proposed model is considered essential. This adjustment is particularly critical for ensuring consistent performance across varying training conditions.

**Figure 4:**
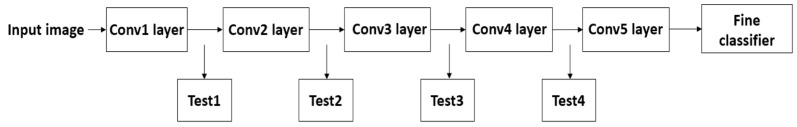
The test placement of coarse classifier in the proposed model.

Moreover, it is important to analyze the effect of the coarse classifier’s placement within the proposed model on overall classification accuracy. As shown in Figure 4, four different positions are tested, referred to as Test1, Test2, Test3, and Test4. According to the results in Table 5, the coarse classifier at Test1 and Test2 exhibits suboptimal performance due to the limited number of layers and convolutional kernels, which in turn leads to reduced accuracy in the fine classification stage. Although Test4 benefits from a deeper architecture, its proximity to the fine classifier reduces the effectiveness of the coarse classifier in influencing model training via loss weighting, resulting in slightly lower fine classifier accuracy compared to Test3. Therefore, based on overall accuracy improvement, the proposed model adopts Test3 as the optimal placement for the coarse classifier.

By incorporating a hierarchical structure of target classes into the model, the branch CNN leverages structured prior knowledge to enhance the classification process, offering a distinct advantage over traditional CNN models. As shown in Table 6, the inclusion of the branch structure leads to a 5–6% improvement in accuracy compared to the model without the branch component.

Based on the architecture of the proposed B-CNN model, the total number of parameters is approximately 24,000. A detailed breakdown of the model parameters is provided in Table 7.

### 3.2. B-CNN with Early Exit Mechanism

**Figure 5:**
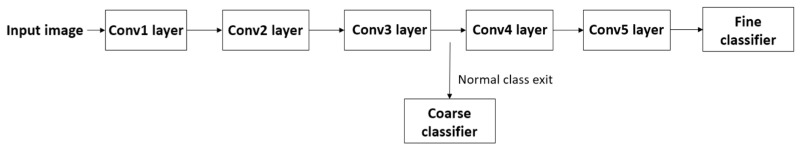
The early exit of Normal class through coarse classifier in proposed model.

Drone-captured images predominantly fall under the Normal class. Therefore, an early-exit mechanism for the Normal class is implemented within the coarse classifier of the proposed model, as illustrated in Figure 5.

Table 8 shows that the coarse classifier achieves lower accuracy than the fine classifier for the Normal class but still maintains an accuracy exceeding 85%. However, the coarse classifier requires fewer parameters and incurs lower computational overhead. As a result, the proposed branch CNN model enables fast inference and energy efficiency by implementing an early-exit mechanism for the Normal class at the coarse classification stage, while utilizing the fine classifier to accurately predict other disaster or accident categories. This design allows drones with limited hardware resources to perform disaster detection more effectively.

### 3.3. Model Quantization Method

Unlike BNN and TWN, which restrict weight quantization to 1-bit or 2-bit precision, DoReFa-Net provides a quantization scheme that supports flexible bit-width selection. Accordingly, DoReFa-Net is adopted in this study for weight quantization. It defines a quantization function that transforms continuous weight values into discrete values ranging between –1 and 1. This section investigates the selection of an appropriate bit-width to strike a balance between memory efficiency and weight precision.

First, it is necessary to determine the number of quantization bits required to represent weight values within the range [−1, 1]. Table 9 presents the classification accuracy of the proposed model under various quantization bit-widths for each classifier. When weights are quantized to 7 bits, only a slight degradation in accuracy is observed. However, reducing the bit-width to 6 bits or fewer results in a significant drop in accuracy. Therefore, the proposed model is configured to quantize weights to 7 bits.

To accelerate model computation, a direct fixed-point representation is employed and stored in memory, rather than using a lookup table for conversion. Since DoReFa-Net constrains weight values to the range [−1, 1], 2 bits are allocated for the integer part of the weights. The number of fractional bits is then determined. As shown in Table 10, allocating 6 bits for the fractional part yields stable accuracy, whereas reducing the fractional precision to 5 bits or fewer leads to a noticeable decline in performance. Thus, each weight is represented using 8 bits in total, with 2 bits assigned to the integer part and 6 bits to the fractional part.

Nevertheless, using DoReFa-Net for activation quantization requires additional multiplication and division operations in hardware implementations, resulting in a significant increase in computational complexity during model inference. To address this issue, the use of PACT for activation quantization is explored. Unlike ReLU, which lacks an upper bound and therefore demands a wide precision range to accurately represent activation values, PACT dynamically learns an upper bound for each layer’s activation during training via backpropagation. Each layer is associated with a distinct upper bound parameter, denoted as *α*. However, this design introduces varying memory requirements for feature maps across layers, thereby posing challenges for efficient memory reuse in subsequent hardware implementations.

As shown in Table 11, although the *α* values for each layer exhibit slight variation between the 100th and 200th training epochs, the resulting accuracy reduction is minimal, ranging from 0.03% to 0.09%. Furthermore, the integer part of the α values across layers falls within the range of 1 to 3. Based on these observations, the proposed method first employs PACT to train the model for a limited number of epochs to determine the activation upper bounds for each layer, and then selects the maximum observed value as a unified upper bound for all layers in the subsequent retraining phase. In this case, the maximum *α* value is 3, which is used as the fixed upper bound across all layers during retraining.

As shown in Table 12, while the fixed-*α* model incurs a slight accuracy reduction of 0.07% to 0.1% compared to the original PACT configuration, it significantly reduces the complexity of subsequent hardware implementation.

Since the activation upper bound is set to 3 for each layer, the integer part is encoded using 2-bit unsigned integers. Table 13 presents the model accuracy across different levels of fractional precision. The results indicate that with 6 bits of fractional precision, the accuracy remains relatively stable. However, a noticeable degradation in accuracy occurs when the fractional precision is reduced to 5 bits. Consequently, the fractional precision is fixed at 6 bits, allowing activation values to be stored using only one-quarter of the memory required by standard 32-bit floating-point representation.

## 4. Hardware Implementation

### 4.1. Fixed-Point Conversion of Batch Normalization

Upon completion of model training, each batch normalization layer produces four parameters: the mini-batch mean ($\mu_{B}$), mini-batch variance ($\alpha_{B}^{2}$), and the learnable scaling (*γ*) and shifting (*β*) factors. To reduce the computational complexity in hardware implementation, these four parameters can be algebraically reduced to two simplified terms using mathematical formulations, as shown in Equations (3) and (4), where $\mu_{B}$ denotes the mini-batch mean and $\alpha_{B}^{2}$ the mini-batch variance. As a result, the batch normalization computation can be efficiently implemented using only multiplication and addition operations, as illustrated in Equation (5).

(3) $$\gamma^{\prime }=\frac{1}{\sqrt{\alpha_{B}^{2}}}\gamma$$

(4) $$\beta^{\prime }=-\left(\mu_{B}\gamma^{\prime }\right)+\beta$$

(5) $$y_{i}=\gamma^{\prime }x_{i}+\beta^{\prime }$$

Next, the simplified batch normalization parameters are subject to fixed-point quantization. Taking *γ′* as an example, since its values lie within the range [0, 1], 2 bits are allocated for the integer part. The fractional bit-width is then determined based on quantization sensitivity. As shown in Table 14, when the fractional precision ranges from 9 to 11 bits, model accuracy remains largely unaffected. However, a noticeable decline in accuracy is observed when the fractional precision is reduced to 8 bits or fewer. Therefore, each *γ′* value is represented using 11 bits in total, comprising 2 bits for the integer part and 9 bits for the fractional part.

After completing the fixed-point quantization of *γ′*, a similar procedure is applied to *β′*. Since *β′* values range from −3 to 3, 3 bits are allocated for the signed integer part. The next step involves determining the appropriate fractional precision. As shown in Table 15, the model maintains stable accuracy when the number of fractional bits ranges from 6 to 8. However, a significant degradation in accuracy is observed when the fractional precision is reduced to 5 bits or fewer. Therefore, *β′* is represented in fixed-point format using 3 integer bits and 6 fractional bits.

### 4.2. B-CNN Hardware Accelerator Architecture

**Figure 6:**
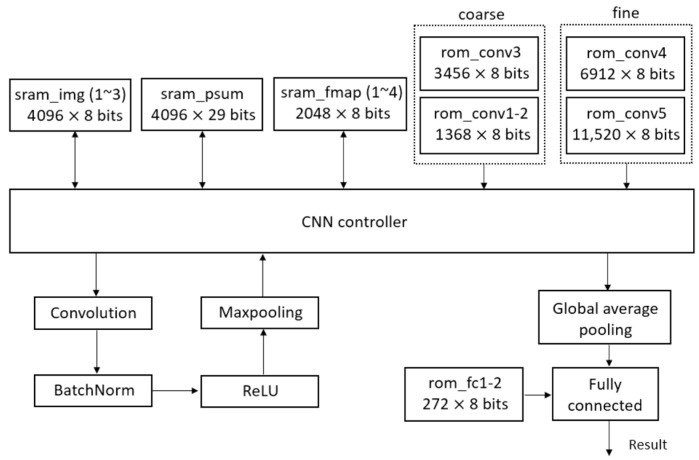
The hardware architecture of proposed B-CNN model with early exit mechanism.

This section presents a detailed discussion of the hardware implementation strategy and memory utilization. To achieve high efficiency and flexibility, the proposed hardware architecture utilizes fully on-chip memory blocks, eliminating the requirement for external memory access. This design choice maximizes memory bandwidth utilization and enables improved control over memory access scheduling and power management. At the software-level optimization stage, memory consumption is carefully minimized by compressing selected intermediate features (as described in detail in Section 3). Furthermore, memory usage is partitioned into multiple smaller blocks tailored specifically to the demands of each network layer, thus enabling finer-grained activation control and reducing overall energy consumption. In addition, to decrease system complexity and power consumption, convolution computations are implemented using a single set of processing elements (PEs), which are reused across different layers through a time-multiplexed mechanism. Figure 6 illustrates the hardware architecture of the proposed B-CNN model, which incorporates an early-exit mechanism.

The memory requirements for hardware computation are primarily divided into two types: Static Random-Access Memory (SRAM) and Read-Only Memory (ROM). SRAM is utilized to store the input image, intermediate partial sums generated during computation, and the feature maps produced after each convolutional layer. ROM is used to store the weights associated with the convolutional and fully connected layers.

The convolution block performs zero-padding on the input image and feature maps, and applies convolution operations using the corresponding weights. The batch normalization block processes the convolution outputs using the parameters *γ′* and *β′*, as defined in Equation (5), through multiplication and addition operations. These parameters are stored in a look-up table, eliminating the need for an additional ROM. The ReLU block replaces negative values with zero. The max pooling block performs the pooling operation to reduce the spatial dimensions of the feature maps. Finally, the global average pooling block computes the average across each input channel and forwards the results to the fully connected block for final model prediction.

Table 16 summarizes the memory usage for each layer. The input is a 64 × 64 RGB image, with each pixel value represented using 8 bits. This image is stored across three SRAM modules, namely sram_img1, sram_img2, and sram_img3, amounting to 98,304 bits. This constitutes the largest memory allocation in the entire computation process.

After loading the input image into SRAM, convolution operations begin. The feature map generated by the first convolutional layer is stored in four SRAM modules: sram_fmap1, sram_fmap2, sram_fmap3, and sram_fmap4, with a total memory usage of 65,536 bits. This makes it the largest intermediate feature map in the model. Due to max pooling, the spatial resolution of feature maps is halved in each subsequent layer. Consequently, from the third layer onward, feature maps are alternately stored in sram_fmap1 and sram_fmap2, eliminating the need for additional memory allocation.

**Figure 7:**
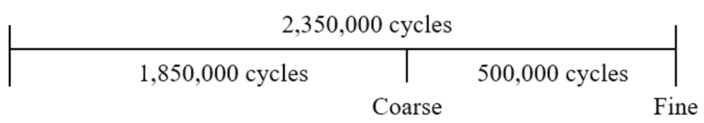
The different cycle counts for coarse and fine prediction.

The proposed B-CNN model comprises a coarse prediction stage followed by a fine prediction stage. The cycle count required to complete the full model inference is illustrated in Figure 7. Inference with the B-CNN model requires a total of 2,350,000 cycles. However, the coarse prediction stage alone completes 1,850,000 cycles. This enables early termination of inference for data belonging to the Normal class, significantly reducing computational demand and power consumption.

For other categories such as disasters or accidents, more extensive computation is required to ensure higher classification accuracy. Nevertheless, after the coarse classifier, only an additional 500,000 cycles are required to complete the fine prediction stage.

Power gating is a key technique in memory management, aimed at improving energy efficiency in electronic systems. By selectively disabling power to inactive sections of a circuit, it significantly reduces both dynamic and static power consumption. In memory systems, energy savings are achieved by deactivating unused memory blocks, ensuring that only the memory blocks required for current operations remain powered.

**Figure 8:**
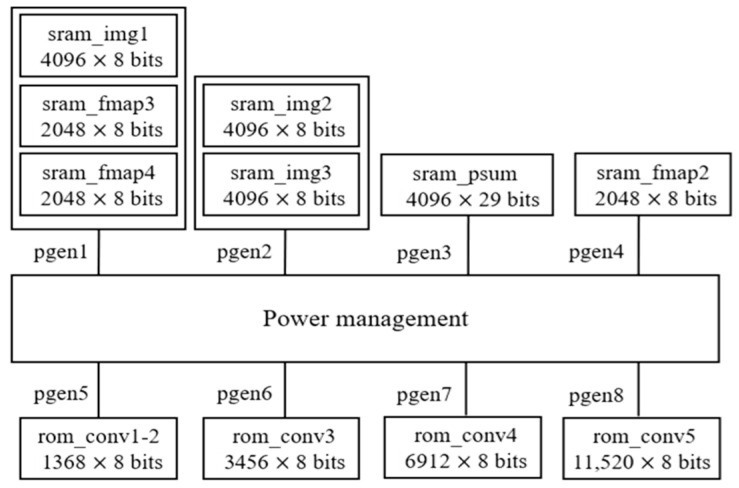
The power management for memory power gating control.

Control circuitry plays a critical role in managing the power supply by determining when to enable or disable specific memory regions based on access patterns. This technique not only enhances energy efficiency but also reduces heat generation, which is essential for maintaining device reliability and prolonging operational lifespan. The proposed memory power gating strategy is illustrated in Figure 8. Notably, in the proposed design, power gating is applied exclusively to the memory subsystem, such as ROM and RAM modules. Other components, including logic and control circuits, are not subjected to power gating in this architecture.

The power control module, referred to as the power management unit, generates control signals (pgen1 to pgen8) to operate the power switches associated with various memory blocks. Due to shared circuit activity, sram_img1, sram_fmap3, and sram_fmap4 are managed collectively by the control signal pgen1, while sram_img2 and sram_img3 are controlled by pgen2. Additional details regarding memory power management across the entire hardware architecture are provided in Table 17.

**Figure 9:**
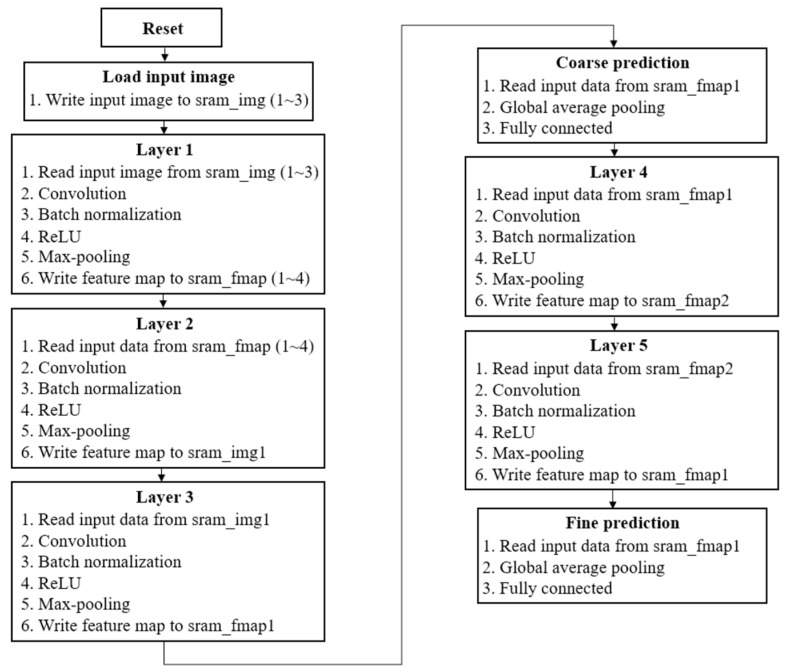
The computation flow for the proposed B-CNN hardware design.

Figure 9 illustrates the computation flow of the proposed B-CNN hardware architecture. After loading the input image, the inference process is divided into a three-layer coarse prediction stage or a five-layer fine prediction stage. Each layer performs a similar sequence of operations, including convolution, batch normalization, ReLU activation, max-pooling, and memory read/write processes. The final prediction result, regardless of whether it is produced by the coarse or fine prediction stage, is obtained by applying global average pooling to the feature map, followed by a fully connected layer.

## 5. Experimental Results

This section presents an analysis of the experimental results for the proposed B-CNN hardware design. The design was implemented using TSMC 16 nm CMOS technology. To evaluate power consumption, an automatic placement and routing (APR) flow was employed to collect relevant experimental data. The layout was completed using Cadence Innovus 21.17, followed by post-layout simulation to generate switching activity data. Subsequently, the power consumption was estimated using the Powermeter utility within Cadence Voltus 21.16, incorporating simulated switching activity and standard cell library power models. Interconnect parasitics, including resistance and capacitance (RC) effects, were also taken into account. During the post-layout simulation, the design operated at a frequency of 500 MHz. Note that, the proposed design’s maximum clock frequency (500 MHz) is limited by the ROM access time, with rom_conv5 being the critical path due to its relatively long delay.

**Figure 10:**
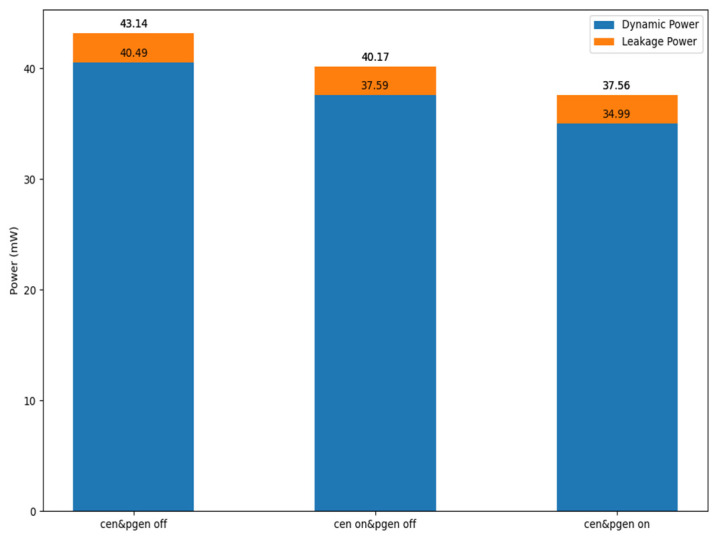
The power management for power gating control.

As shown in Figure 10, the proposed B-CNN hardware incorporating power-gating operates at a power consumption of 37.56 mW under a 500 MHz clock frequency. This corresponds to a reduction of approximately 12.9% compared to the design without power-gating. These results demonstrate the efficacy of the power-gating technique in achieving low-power operation for the circuit.

Table 18 illustrates the classification accuracy at various stages, from software implementation to hardware realization. The results indicate that, following quantization and fixed-point representation, the representable numerical precision of model parameters is reduced, leading to a cumulative accuracy degradation of approximately 2.1%. During the Verilog Register Transfer Level (RTL) simulation phase of the proposed B-CNN hardware, minor bit-level discrepancies in computation further contributed to the reduction in accuracy. Nevertheless, the overall classification accuracy remains above 88%.

Table 19 summarizes the test accuracy and classification results obtained during the Verilog RTL simulation stage. Following model quantization and fixed-point conversion, the highest classification accuracy is observed for the Normal category, reaching 93.17% and remaining above 90%. In contrast, the lowest accuracy is recorded for the Traffic Accident category, with a classification accuracy of only 73.20%. To further analyze the system behavior, missed detection and false alarm rates were calculated based on the experimental results. The missed detection rate was approximately 8.58%, indicating that some disaster scenarios were incorrectly classified as normal. The false alarm rate was approximately 5.47%, suggesting that normal scenarios were mistakenly identified as disasters. Despite these errors, their impacts can be mitigated through repeated detection of spatially or temporally adjacent frames. Such redundancy-based strategies are particularly suitable for drone-based applications, as video inputs from these applications provide continuous observations of the same area.

Following multiple adjustments and testing, the proposed B-CNN hardware circuit achieves a maximum operating frequency of 500 MHz. As shown in Equation (6), the total inference time for a complete model prediction is 0.0047 s. Equations (7) and (8) detail the computation times for each classifier, with the coarse classifier requiring 0.0037 s and the fine classifier requiring 0.001 s. It is worth noting that the reported latency in this work already accounts for the activation and deactivation overhead introduced by the power gating mechanism. This design enables UAVs to execute disaster detection tasks more rapidly and efficiently, while conserving power during operation.

(6) 2,350,000×2 ns=4,700,000 ns≈0.0047 s

(7) 1,850,000×2 ns=3,700,000 ns≈0.0037 s

(8) 500,000×2 ns=1,000,000 ns≈0.001 s

**Figure 11:**
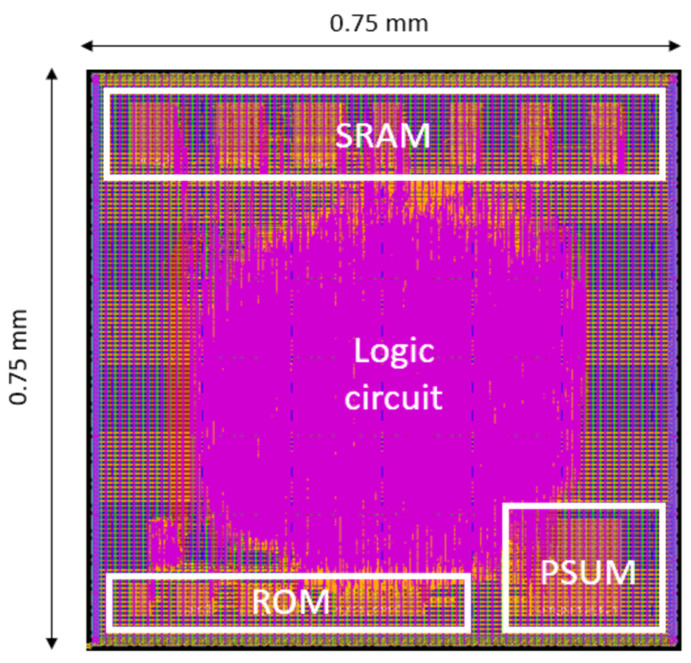
The layout of the proposed B-CNN circuit.

The chip layout of the proposed B-CNN hardware design is shown in Figure 11, which illustrates the locations of the ROM, SRAM, and partial sum (PSUM) components. The total chip area is 0.75 × 0.75 mm^2^. Note that Figure 11 illustrates a verification layout used for post-layout simulation and does not represent a complete tape-out-ready design.

The implementation results of the proposed B-CNN hardware design are summarized in Table 20. The design, implemented using TSMC 16 nm CMOS technology, achieves a maximum operating frequency of 500 MHz, with a power consumption of 37.56 mW and a total memory size of 57 KB. For more detail, the power consumption can be further broken down into memory blocks and logic circuits. The memory blocks include both SRAM and ROM, while the logic circuits refer to the computational components. The power consumption of the memory blocks is approximately 2.169 mW, whereas that of the logic circuits is around 35.39 mW.

Table 21 provides a detailed comparison of prior arts used for processing the AIDER dataset, including various hardware information, image sizes, quantization methods, inference times, memory sizes, FLOPs, and accuracies.

Since the power consumption details in references [9,10,11,12] are not reported, the power estimates are derived primarily from manufacturer specifications: 91 W for the Intel i7, 28 W for the Intel i5, and 70 W for the NVIDIA T4 GPU. Additionally, memory usage is not provided in reference [12]; only the number of model parameters is reported. As a result, the comparison table assumes each parameter is represented as a 32-bit floating-point value and converts this into kilobytes for estimation. Furthermore, the accuracy of 83.1% attributed to reference [10] is derived from the analysis presented in reference [12].

The proposed B-CNN outperforms the models in [9,10,11,12] in terms of inference time and memory efficiency by resizing input images to 64 × 64 pixels and employing model quantization to reduce both the number of parameters and their bit-width. This enables faster predictions with lower memory requirements in hardware implementation. Although the proposed model exhibits slightly lower accuracy compared to [9,12] due to reduced parameter complexity, the incorporation of branch-based training strategies enhances feature learning, resulting in competitive overall performance.

## 6. Conclusions

This paper proposes a B-CNN model with an early-exit mechanism for UAV-based disaster detection. A label tree is constructed to address frequently confused classes, enhancing feature representation through branch-based training and reducing the parameter count with negligible loss in classification accuracy. The model employs DoReFa-Net along with a clipped activation function to constrain weights and activations, while fixed-point quantization is applied to determine appropriate decimal precision. Software evaluation shows that the B-CNN achieves 90.21% accuracy with only 24K parameters. After quantization, it maintains an accuracy of 88.38% using 8-bit weights and activations, reducing memory and computational demands to just 25% of those required by conventional 32-bit CNNs. Batch normalization is simplified using mathematical expressions, allowing computation with only two parameters via basic arithmetic operations, followed by fixed-point quantization.

For hardware implementation, memory power consumption is reduced through power gating by disabling idle memory blocks. The proposed hardware design is implemented using TSMC 16 nm CMOS technology, operates at 500 MHz, consumes 37.56 mW of power, and achieves a final classification accuracy of 88.18%.

Moreover, since UAVs often operate in complex environments, they may encounter various challenging conditions such as fog, nighttime scenes, or low visibility. To address these issues, several image enhancement techniques have been proposed in the literature. For example, Sun et al. [19] introduced an adaptive dehazing and tracking framework that selectively performs dehazing during hazy conditions, improving tracking robustness in UAV videos. Similarly, Liu et al. [20] proposed a variational nighttime image dehazing method tailored for intelligent transportation systems, which effectively enhances visibility in dark and glowing scenes using hybrid regularization. In the future, we plan to explore the integration of such lightweight image preprocessing techniques into our system, allowing our design to better adapt to challenging environmental conditions and further enhance its practical applicability.

## Figures and Tables

**Table 1:** The amount of training set and test set in each class.

Class	Amount of Training Data	Amount of Test Data
Fire	417	104
Flood	421	105
Collapsed building	409	102
Traffic accidents	388	97
Normal	3512	878
Total	5147	1286

**Table 2:** The accuracy of different compression methods across various image sizes.

Image Size	Nearest Neighbor Interpolation Test Accuracy	Bilinear Interpolation Test Accuracy	Area Interpolation Test Accuracy	Bicubic Interpolation Test Accuracy
128 × 128	85.31%	90.28%	90.34%	90.31%
64 × 64	78.72%	90.12%	90.21%	90.25%
32 × 32	66.34%	73.57%	74.39%	75.44%

**Table 3:** The confusion matrix for creating the label tree.

	Fire	Flood	Collapsed Building	Traffic Accidents
Fire	75	25	3	1
Flood	25	67	6	7
Collapsed building	6	11	47	38
Traffic accidents	8	6	27	56

**Table 4:** The test accuracy of the branch CNN model with and without BN.

	With Batch Normalization	Without Batch Normalization
Test accuracy	90.21%	86.67%

**Table 5:** The test accuracy of the placement of coarse classifier in the proposed model.

	Test1	Test2	Test3	Test4
Test accuracy	85.57%	87.85%	90.21%	90.07%

**Table 6:** The test accuracy of the branch CNN model with and without branch.

	With Branch	Without Branch
Test accuracy	90.21%	84.67%

**Table 7:** The number of parameters of each operation.

Operation	Number of Parameters	Sum of Parameters
CONV	3 × 3 × 3 × 8	216
BatchNorm	4 × 8	32
CONV	8 ×3 × 3 × 16	1152
BatchNorm	4 × 16	64
CONV	16 × 3 × 3 × 24	3456
BatchNorm	4 × 24	96
FC	24× 3	72
CONV	24 × 3 × 3 × 32	6912
BatchNorm	4 × 32	128
CONV	32 × 3 × 3 × 40	11,520
BatchNorm	4 × 40	160
FC	40 × 5	200
Total parameters:		24,008

**Table 8:** The comparison of coarse and fine classifier to the Normal class.

	Parameters	FLOPs	Accuracy
Coarse classifier	5152	6.1 M	88.67%
Fine classifier	24,008	7.4 M	95.32%

**Table 9:** The accuracy of weight quantization with different bits in DoReFa-Net.

Bits	Coarse Accuracy	Fine Accuracy
FP32	87.53%	90.21%
9	87.21%	89.95%
8	87.05%	89.87%
7	86.81%	89.72%
6	85.23%	88.48%
5	83.40%	86.28%
4	63.18%	70.36%

**Table 10:** The accuracy of weight quantization with different decimal bits.

Integer Bits	Decimal Bits	Coarse Accuracy	Fine Accuracy
2	9	86.78%	89.68%
2	8	86.75%	89.67%
2	7	86.41%	89.51%
2	6	86.27%	89.43%
2	5	84.75%	88.39%

**Table 11:** The alpha value in each layer with 100 epochs and 200 epochs.

Alpha Value	100 Epochs	200 Epochs
Layer 1	1.3719286	1.3830165
Layer 2	1.3525951	1.3779949
Layer 3	2.6114667	2.6246102
Layer 4	1.1478527	1.1531808
Layer 5	2.0920019	2.0938022
Coarse accuracy	86.21%	86.24%
Fine accuracy	89.27%	89.36%

**Table 12:** The accuracy of setting different alpha value.

	Clipping Value to 3	PACT
Coarse accuracy	86.14%	86.21%
Fine accuracy	89.17%	89.27%

**Table 13:** The accuracy of activation quantization with different decimal bits.

Integer Bits	Decimal Bits	Coarse Accuracy	Fine Accuracy
2	9	86.14%	89.17%
2	8	86.05%	89.15%
2	7	85.85%	89.08%
2	6	85.57%	89.02%
2	5	84.26%	87.68%

**Table 14:** The test accuracy comparison table for the bit-width of $\gamma^{\prime }$.

Integer Bits	Decimal Bits	Coarse Accuracy	Fine Accuracy
2	11	85.48%	88.96%
2	10	85.43%	88.82%
2	9	85.36%	88.69%
2	8	82.75%	87.12%
2	7	77.57%	82.43%
2	6	70.81%	76.53%

**Table 15:** The test accuracy for different bit-width of *β’*.

Integer Bits	Decimal Bits	Coarse Accuracy	Fine Accuracy
3	8	85.34%	88.65%
3	7	85.25%	88.54%
3	6	85.12%	88.38%
3	5	83.75%	87.43%
3	4	78.69%	82.43%

**Table 16:** The memory usage table for each layer.

Stored Data	Feature Map Size (Width × Height × Channel × Bit-Width)	Total Size (Bits)	Data Management
Input image	64 × 64 × 3 × 8	98,304	sram_img1, sram_img2, sram_img3
Feature maps of layer 1	32 × 32 × 8 × 8	65,536	sram_fmap1, sram_fmap2, sram_fmap3, sram_fmap4
Feature maps of layer 2	16 × 16 × 16 × 8	32,768	sram_img1
Feature maps of layer 3	8 × 8 × 24× 8	12,288	sram_fmap1
Feature maps of layer 4	4 × 4 × 32 × 8	4096	sram_fmap2
Feature maps of layer 5	2 × 2 × 40 × 8	1280	sram_fmap1

**Table 17:** The power state in each layer.

Layer	Power Domain							
	pgen1	pgen2	pgen3	pgen4	pgen5	pgen6	pgen7	pgen8
layer1	On	On	On	On	On	Off	Off	Off
layer2	On	Off	On	On	On	Off	Off	Off
layer3	On	Off	On	Off	Off	On	Off	Off
layer4	Off	Off	On	On	Off	Off	On	Off
layer5	Off	Off	Off	On	Off	Off	Off	On

**Table 18:** The test accuracy with different operations.

Operation of Each Stage	Coarse Accuracy	Fine Accuracy
Build B-CNN model	87.53%	90.21%
Weight quantization to 8 bits	86.27%	89.43%
Activation quantization to 8 bits	85.57%	89.02%
Convert γ′ in BN to 11 bits	85.24%	88.63%
Convert β′ in BN to 9 bits	85.02%	88.38%
Verilog Register Transfer Level	84.85%	88.18%

**Table 19:** The test accuracy and classification results in Verilog RTL simulation.

Image Type	Image Number (Correct/Total)	Test Accuracy
Collapsed building	75/102	73.52%
Fire	84/104	80.77%
Flood	86/105	81.90%
Normal	818/878	93.17%
Traffic accident	71/97	73.20%
Overall accuracy	1134/1286	88.18%

**Table 20:** The B-CNN hardware implementation results.

		The Proposed Architecture			
	
Technology		TSMC 16 nm CMOS			
Frequency (MHz)		500			
Power (mW)		Total	37.56	Memory block	2.169
				Logic circuit	35.39
Chip area (mm^2^)		0.5625			
Parameters		24,008			
Memory size (KB)		57			

**Table 21:** Comparison with prior methods.

	IEEE CVPR’2019 [9]	IEEE JSTARS’2020 [10]	IEEE EUVIP’2022 [11]	IEEE LGRS’2023 [12]	Proposed Work
Dataset	AIDER	AIDER	AIDER	AIDER	AIDER
Architecture	ERNet	EmergencyNet	CNN	WATT-EffNet	Branch CNN
Hardware information	Intel i7	ARM Cortex-A57	Intel i5	NVIDIA Tesla T4 GPU + TPU	16 nm ASIC
Image size	240 × 240	240 × 240	100 × 100	224 × 224	64 × 64
Quantization method	No	No	No	No	DoReFa-Net + Fixed-point
Inference time (s)	0.018	0.041	0.069	N/A	0.0037 (coarse)/ 0.0047 (fine)
Memory size (KB)	300	360	3072	2690	57
FLOPs	N/A	57 M	22 M	N/A	6.1 M (coarse)/ 7.4 M (fine)
Accuracy	90.1%	83.1%	88%	88.5%	90.21% (software)/ 88.18% (hardware)

Note: N/A = not available, indicating that data were not provided by the comparative references.

## Data Availability

The original contributions presented in this study are included in the article. Further inquiries can be directed to the corresponding author.

## References

[1] Pham H., Ichalal D., Mammar S. Complete coverage path planning for pests-ridden in precision agriculture using UAV. Proceedings of the IEEE International Conference on Networking, Sensing and Control (ICNSC).

[2] Wang S., Han Y., Chen J., Zhang Z., Wang G., Du N. A deep-learning-based sea search and rescue algorithm by UAV remote sensing. Proceedings of the IEEE CSAA Guidance, Navigation and Control Conference (CGNCC).

[3] Mohd-Tajudin M.-H., Mat-Desa S., Abdullah J. Traffic density identification from UAV images using deep learning: A preliminary study. Proceedings of the International Conference on Software, Knowledge, Information Management and Applications (SKIMA).

[4] Shubhasree A.V., Sankaran P., Raghu C.V. UAV image analysis of flooded area using convolutional neural networks. Proceedings of the International Conference on Connected Systems & Intelligence (CSI).

[5] Vu H.N., Nguyen H.M., Pham C.D., Tran A.D., Nguyen Trong K., Pham C., Nguyen V.H. Landslide detection with unmanned aerial vehicles. Proceedings of the International Conference on Multimedia Analysis and Pattern Recognition (MAPR).

[6] Shamsoshoara A., Afghah F., Razi A., Zheng L., Fulé P.Z., Blasch E. (2021). Aerial imagery pile burn detection using deep learning: The FLAME dataset. Comput. Netw..

[7] DJI Drones. https://store.dji.com/tw/content/long-range-drone.

[8] Jetson Nano Devkit Datasheet. https://developer.nvidia.com/embedded/downloads.

[9] Kyrkou C., Theocharides T. Deep-Learning-Based Aerial Image Classification for Emergency Response Applications using Unmanned Aerial Vehicles. Proceedings of the Computer Vision and Pattern Recognition Workshops.

[10] Kyrkou C., Theocharides T. (2020). EmergencyNet: Efficient aerial image classification for drone-based emergency monitoring using atrous convolutional feature fusion. IEEE J. Sel. Top. Appl. Earth Obs. Remote Sens..

[11] Munsif M., Afridi H., Ullah M., Khan S.D., Cheikh F.A., Sajjad M. A lightweight convolution neural network for automatic disasters recognition. Proceedings of the European Workshop on Visual Information Processing (EUVIP).

[12] Lee G.Y., Dam T., Ferdaus M.M., Poenar D.P., Duong V.N. (2023). WATT-EffNet: A lightweight and accurate model for classifying aerial disaster images. IEEE Geosci. Remote Sens. Lett..

[13] Courbariaux M., Hubara I., Soudry D., El-Yaniv R., Bengio Y. (2016). Binarized neural networks: Training deep neural networks with weights and activations constrained to +1 or −1. arXiv.

[14] Li F., Liu B., Wang X., Zhang B., Yan J. (2022). Ternary weight networks. arXiv.

[15] Choi J., Wang Z., Venkataramani S., Chuang P.I.-J., Srinivasan V., Gopalakrishnan K. (2018). PACT: Parameterized clipping activation for quantized neural networks. arXiv.

[16] Zhou S., Wu Y., Ni Z., Zhou X., Wen H., Zou Y. (2018). DoReFa-Net: Training low bitwidth convolutional neural networks with low bitwidth gradients. arXiv.

[17] Zhu X., Bain M. (2017). B-CNN: Branch convolutional neural network for hierarchical classification. arXiv.

[18] Teerapittayanon S., McDanel B., Kung H.T. BranchyNet: Fast inference via early exiting from deep neural networks. Proceedings of the International Conference on Pattern Recognition (ICPR).

[19] Sun L., Chang J., Zhang J., Fan B., He Z. (2023). Adaptive Image Dehazing and Object Tracking in UAV Videos Based on the Template Updating Siamese Network. IEEE Sens. J..

[20] Liu Y., Wang X., Hu E., Wang A., Shiri B., Lin W. (2025). VNDHR: Variational Single Nighttime Image Dehazing for Enhancing Visibility in Intelligent Transportation Systems via Hybrid Regularization. IEEE Trans. Intell. Transp. Syst..

